# Targeting STAT3 for Cancer Therapy: Focusing on Y705, S727, or Dual Inhibition?

**DOI:** 10.3390/cancers17050755

**Published:** 2025-02-23

**Authors:** Kyli Berkley, Julian Zalejski, Ashutosh Sharma

**Affiliations:** Department of Chemistry, University of Illinois Chicago, Chicago, IL 60607, USA; kberk4@uic.edu (K.B.); jzalej2@uic.edu (J.Z.)

**Keywords:** STAT3, cancer therapy, STAT3 inhibitor, Y705, S727

## Abstract

Background/Objectives: Signal Transducer and Activator of Transcription 3 (STAT3) is a transcription factor that is strongly implicated in various cancers. In its canonical signaling pathway, Janus kinases (JAKs) phosphorylate STAT3 at the Y705 residue in response to cytokines or growth factors, with pY705 serving as a key marker of STAT3 oncogenic activity. Elevated pY705 levels correlate with poor prognosis, and numerous small-molecule inhibitors have been developed to block this phosphorylation site. More recently, phosphorylation at the S727 residue (pS727) has emerged as a critical contributor to STAT3-mediated oncogenesis, particularly due to its role in mitochondrial translocation. Evidence suggests that pS727 may even surpass pY705 in driving oncogenic activity. These findings prompt an important question: Which residue should be prioritized for effective STAT3 inhibition in cancer therapy? Methods: This review compiles and critically analyzes the current literature on STAT3 inhibitors targeting pY705 and/or pS727, evaluating their therapeutic efficacy in vitro, in vivo, and in clinical trials. We assess the unique effects of targeting each residue on downstream signaling, toxicity, and clinical outcomes. Results: Our analysis indicates that inhibitors targeting both pY705 and pS727 achieve the greatest therapeutic effectiveness. However, pS727 targeting is associated with higher toxicity risks. Conclusions: Comprehensive evaluation of STAT3 inhibitors underscores the importance of targeting pY705 for maximum therapeutic benefit. The analysis also shows that co-targeting pS727 may increase overall efficacy. However, pS727 inhibition should be approached with lower affinity to minimize toxicity and enhance the clinical feasibility of dual-targeting strategies.

## Simple Summary

Signal Transducer and Activator of Transcription 3 (STAT3) is a promising drug target for cancer therapy. Traditionally, STAT3 inhibitors have focused on blocking phosphorylation at the Y705 residue. However, recent studies reveal that phosphorylation at the S727 residue also plays a critical role in cancer progression. This raises the question of which residue should be prioritized for therapeutic development. In this review, we evaluate the current STAT3 inhibitors and discuss the implications of targeting Y705, S727, or both residues for safe and effective cancer treatment.

## 1. Introduction

Signal transducer and activator of transcription 3 (STAT3) is a transcription factor that plays a pivotal role in various cellular processes, including cell growth, differentiation, survival, and immune responses [1,2]. Normally, STAT3 activation is transient and tightly regulated in healthy cells; however, in many cancers, STAT3 is constitutively active thus promoting cell proliferation, invasion, drug resistance, and immune cell evasion [1,3,4,5,6]. Basal-level STAT3 signaling is necessary for regular cell maintenance, as evidenced by the fact that STAT3 knockouts proved lethal in embryonic mouse studies [7,8]. However, in cancer, hyperphosphorylated STAT3 activates oncogenic transcription factors such c-Myc and Oct-4, leading to enhanced cell proliferation and survival, as well as aggressive cancer cell stemness [9,10,11]. This aberrant activation has established STAT3 as a critical target for cancer therapy due to its involvement in tumor progression and association with poor disease prognosis.

**Figure 1:**
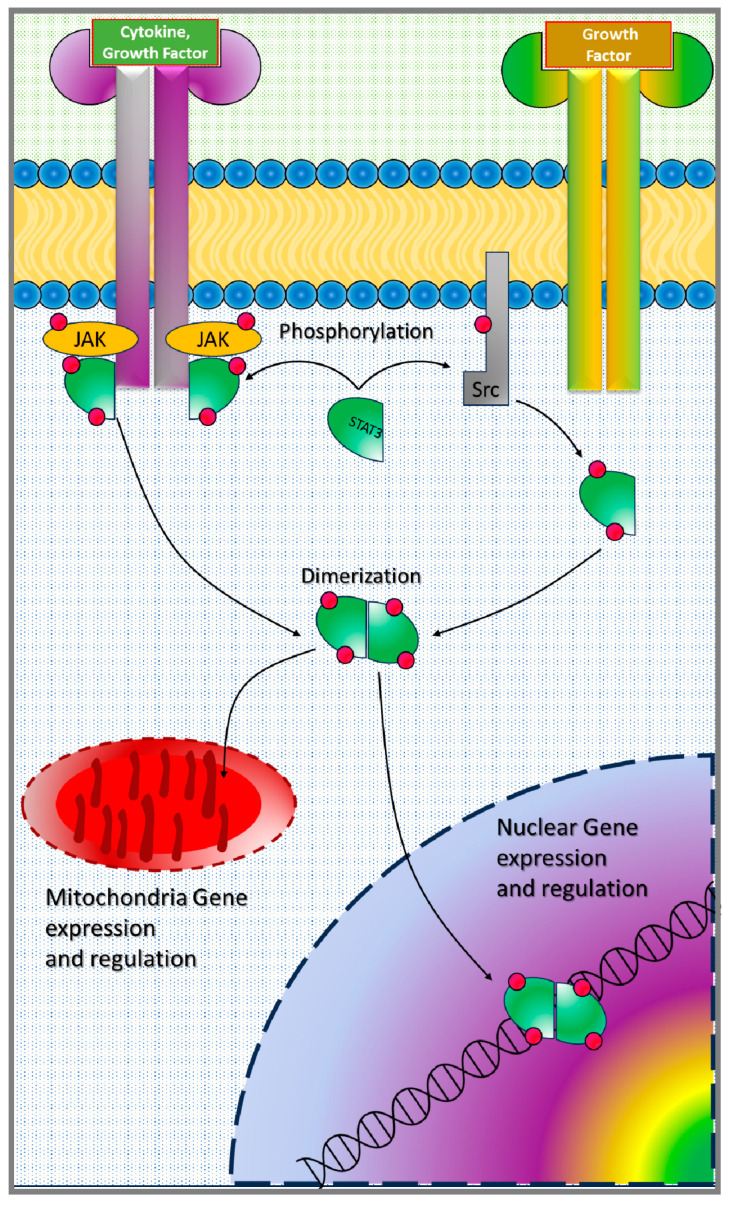
Schematic representation of STAT3 activation in cells.

Inactive STAT3 proteins are dispersed in the cytoplasm until their recruitment to the plasma membrane in response to cytokine or growth factor signals, where they are phosphorylated by upstream protein kinases such as Janus kinase proteins (JAKs), proto-oncogene tyrosine-protein kinase Src (Src), and mitogen-activated protein kinases (MAPKs) (Figure 1) [12,13]. Once phosphorylated at the Y705 site, STAT3 dimerizes with itself or other STAT family members and travels to the nucleus, where it initiates gene transcription [14]. Recent studies have shown that S727 also undergoes phosphorylation and is necessary for mitochondrial translocation, but whether or not pS727 should be inhibited to improve cancer therapy remains debated [15,16,17,18,19].

Due to its central role in oncogenesis, STAT3 has garnered significant interest as a therapeutic target. Numerous efforts have been made over the last twenty years to develop small molecules to inhibit the STAT3 signaling axis; however, none have reached FDA approval for cancer treatment, underscoring the need for a better understanding of oncogenic STAT3 signaling mechanisms in order to develop an effective therapeutic approach [1,6,9,20]. Small molecule inhibitors, such as napabucasin and OPB-51602, aim to block STAT3 activation directly or indirectly. Additionally, antisense oligonucleotides, peptides, and other modalities are being investigated for STAT3 inhibition. Current research also suggests selectively targeting Y705 or S727, or both, could differentially affect downstream oncogenic signaling. The dual activation of STAT3 at pY705 and pS727 allows STAT3 to promote oncogenic signaling both in the nucleus and in mitochondria. However, there have been mixed results as to whether both pY705 and pS727 should be targeted for effective cancer treatment [21,22]. Thus, the purpose of this review is to synthesize the current findings in this field to inform current STAT3 therapeutic development with respect to pY705 versus pS727 inhibition.

## 2. STAT3 Signaling Mechanisms

### 2.1. Canonical STAT3 Signaling and the Role of Y705 Phosphorylation

To initiate downstream signaling, STAT3 is activated in response to extracellular signals, such as cytokines (e.g., IL-6, IL-10) and growth factors (e.g., EGF), through the Janus kinase (JAK) pathway. Latent STAT3 molecules are dispersed in the cytoplasm prior to activation [11]. Upon ligand binding to cytokine receptors, JAK proteins phosphorylate STAT3 at the tyrosine-705 (Y705) residue, prompting dimerization, nuclear translocation, and subsequent binding to DNA sequences to initiate transcription of the target genes involved in cell cycle progression and survival. While STAT3 activation can occur in response to various cytokines and growth factors, interleukin-6 (IL-6) is the most well-known activator of STAT3 signaling [23,24]. IL-6 binds to the IL-6 receptor-α (IL-6Rα) on the cell surface which induces conformational changes, leading to the formation of a hexameric signaling complex, including a gp130 homodimer and two IL-6-IL-6Rα heterodimers [23]. This results in Janus kinase (JAK) activation, which are tyrosine kinases that are constitutively associated with the proline-rich cytoplasmic domain of gp130 near the plasma membrane [13,23]. In total, there are four members of the JAK family—JAK1, JAK2, JAK3, and TYK2 [25]. Then, the activated JAKs cause gp130 to become phosphorylated, which recruits cytosolic STAT3 for phosphorylation. This STAT3 signaling cascade can also be initiated by epidermal growth factor (EGF) and interleukin-11 (IL-11) [24,26]. In all, the JAK-STAT pathway can be activated by over 50 different cytokine receptors, leading to the modulation of cell cycle progression, immune response, and inflammation [25,27].

While JAK-mediated signaling can affect any of the STAT protein family members (STAT1, STAT2, STAT3, STAT4, STAT5α, STAT5β, or STAT6), STAT3 has been the most heavily implicated in oncogenesis and cancer progression [4,11,23]. STAT3 signaling is necessary for regular cell maintenance, as evidenced by the fact that STAT3 knockouts proved lethal in embryonic mouse studies [7]. However, in cancer cells, hyperphosphorylated STAT3 activates oncogenic transcription factors such c-Myc, Cyclin-D1, Bcl-xL, VEGF, and Oct-4, leading to enhanced cell proliferation and survival, as well as aggressive cancer cell stemness [9,10,11]. STAT3 also regulates transcription factors Sox2 and NANOG, which contribute to cancer cell stemness, and TWIST1 and Vimentin, which promote the epithelial–mesenchymal (EMT) transition to occur [28,29]. EMT is a hallmark of cancer metastasis and is a necessary transition for cancer cells to undergo in order to travel to distant sites [28,30].

### 2.2. Noncanonical STAT3 Signaling and the Role of S727 Phosphorylation

Even though pY705 has traditionally been measured as the marker of oncogenic STAT3 activities, a growing number of studies have highlighted the importance of serine-727 (S727) phosphorylation, which can enhance STAT3 transcriptional activity and influence mitochondrial functions [15]. S727 phosphorylation occurs through various signaling pathways, including the MAPK, mTOR, and CDK pathways, often in response to stress signals or mitochondrial activity. Unlike Y705 phosphorylation, which primarily promotes transcriptional activity within the nucleus, S727 phosphorylation has been shown to enhance STAT3’s association with mitochondria, where it regulates cellular respiration and metabolic functions. Since pS727 allows for STAT3 translocation to the mitochondria, pS727 affects the electron transport chain and contributes to the Warburg effect on cell metabolism [12,31]. The Warburg effect describes how cancer cells can rewire their metabolism to support high rates of growth and proliferation by relying on high glucose uptake that results in the production of lactate [32].

Recent studies indicate that pS727 may influence STAT3’s oncogenic potential by supporting mitochondrial biogenesis and modulating reactive oxygen species (ROS) production, which can contribute to tumor progression under hypoxic conditions. This mitochondrial role of STAT3, termed “mitochondrial STAT3” or mitoSTAT3, affects cellular bioenergetics, which is essential for the survival and growth of cancer cells in stressful microenvironments. In some cancers, mitoSTAT3 activity driven by pS727 has been linked to increased tumor invasiveness and resistance to apoptosis, particularly in response to chemotherapy. Despite the fact that recent studies have shown that pS727 is necessary for mitochondrial translocation, it is debated whether or not pS727 should be inhibited to improve cancer therapy due to clinical effectiveness and possible toxicities [15,16,17,18,19].

### 2.3. Interplay Between pY705 and pS727 in Cancer

Initially, it was believed that phosphorylation at both the Y705 and S727 sites were necessary for STAT3 activation, and that S727 phosphorylation occurred sequentially after Y705 phosphorylation [33]. However, increasing evidence has shown that S727 can be phosphorylated independently of Y705 and is capable of driving oncogenic activities by itself thus validating it as an independent therapeutic target [15,16,17,34].

Additionally, the phosphorylation of both residues has demonstrated both complementary and antagonistic effects. For example, one study found that both pY705 and pS727 were required for active mitochondrial STAT3 (mitoSTAT3) [17]. Specifically, the phosphorylation of Y705 by JAK proteins was necessary for importing STAT3 inside of the mitochondria while the phosphorylation of S727 by MAPK was needed for transcriptional activity within the mitochondria. In this case, phosphorylation at both residues worked together to yield downstream effects such as stem cell proliferation. MitoSTAT3 has been shown to bind mitochondrial DNA and associate with mitochondrial transcription factor A, which has been implicated in several cancer types including ovarian, endometrial, and cervical cancers [35,36]. Additionally, one study has shown that mitoSTAT3 affects the transcription of mitochondrial transcripts encoding four subunits of the mitochondrial respiratory chain, leading to the enhanced proliferation of mouse embryonic stem cells [37]. Thus, mitoSTAT3 plays an important role in the transcription of mitochondria target genes, as well as cell proliferation.

The idea that S727 phosphorylation occurs following Y705 phosphorylation has been challenged in a study on pancreatic cancer, where it was discovered that pS727 decreases in response to increased phosphorylation at Y705 [38]. This interplay is of great interest for cancer therapeutics as the inhibition of either Y705 or S727 yields distinct biological outcomes. Targeting Y705 alone may reduce STAT3’s nuclear transcriptional activity, while inhibiting S727 phosphorylation could impair the mitochondrial functions critical for cancer cell metabolism. Consequently, dual-targeting strategies, which inhibit both phosphorylation sites, are being explored to comprehensively block STAT3’s oncogenic functions.

## 3. Clinical Relevance of STAT3 in Cancer and Rational for Therapeutic Development

STAT3 is highly implicated in the progression and survival of various cancers, establishing it as a key target in oncology. The signal transducer and activator of transcription (STAT) family consists of six members, of which STAT3 has been the most implicated in cancer progression [4,11,23]. The phospho-tyrosine residue implicated in STAT3 oncogenic activities is conserved within the STAT family since it is necessary for protein activation and dimerization [39]. However, the phospho-serine residue is absent in STAT2 and STAT6. Despite the fact that these residues are well conserved within the STAT family, STAT3 and STAT5 have been predominately implicated in tumorigenesis [40]. However, STAT3 is generally considered to be the more prominent oncogenic target due to its association with a wide range of tumor types. Additionally, the oncogenic role of STAT5 has been shown to be context dependent. One study showed that patient survival was increased for those with tumors displaying active STAT3 and STAT5, while those with only active STAT3 had reduced survival rates [41]. Likewise, another study on myeloid progenitor cells showed that STAT5 had an antagonistic effect on STAT3 function, leading to inhibited differentiation [42].

The aberrant activation of STAT3 has been reported in numerous cancers, including breast, lung, liver, colorectal, pancreatic, and hematological malignancies [1,9]. STAT3’s role in promoting cell proliferation, inhibiting apoptosis, and modulating the immune response makes it central to oncogenesis and tumor progression, particularly in cancers where STAT3 activation correlates with poor prognosis, treatment resistance, and increased metastasis [14,23]. Thus, STAT3 is the most appealing global oncogenic target within the STAT family.

### 3.1. STAT3 Hyperactivation in Various Cancers Is Associated with Poor Prognosis

In many cancers, summarized in Table 1, STAT3 is constitutively activated, either through cytokine receptor signaling or through mutations in upstream pathways [1,21]. For example, in breast cancer, STAT3 is frequently activated by interleukin-6 (IL-6) through the Janus kinase (JAK) pathway, promoting an inflammatory microenvironment that fosters tumor growth [3,4,11]. Zhong et al. showed that STAT3 was upregulated in gastric tumors, as well as in triple-negative breast cancer (TNBC), and that this upregulation correlated with lower survival overall [18]. Several other studies have supported the observation that increased STAT3 activity corresponds with poorer breast cancer prognosis, especially in TNBC [30,43,44,45].

In lung cancer, STAT3 activation is associated with enhanced metastatic potential, aiding in epithelial-to-mesenchymal transition (EMT), which allows cancer cells to spread to distant organs [46]. Due to its role in EMT, constitutively activated STAT3 has been correlated with poor prognosis in non-small-cell lung cancer [47]. In glioblastoma, constitutive STAT3 signaling has been shown to support angiogenesis through the upregulation of vascular endothelial growth factor (VEGF) [48]. Several studies have also shown that upregulated STAT3 contributes to poor prognosis for patients with glioblastoma, especially due to the role STAT3 plays in immune evasion [49,50]. Hepatocellular carcinoma progression has also been shown to be worsened by STAT3 overactivation [51,52]. Additionally, STAT3 hyperactivation has been implicated in cancer progression and poor prognosis in pancreatic cancer [53,54], prostate cancer [55], colorectal cancer [56], and esophageal cancer [57,58]. In all, STAT3 has been shown to be an appealing pan-cancer therapeutic target [59].

### 3.2. Rationale for Targeting STAT3 in Cancer

The following three main signaling axes have been implicated in cancer progression and heavily targeted for therapeutic development: PI3K/AKT, RAS/RAF/MEK, and JAK/STAT. While numerous PI3K inhibitors have been developed, they have been met with various complications upon reaching clinical trials. For instance, pan-PI3K inhibitors, which act on all four PI3K isoforms, have demonstrated a lack of clinical effectiveness, with only copanlisib showing significant effect in clinical trials [60,61]. However, pan-PI3K inhibitors also greatly increase the risk of off-target toxicity. Capivasertib, a pan-AKT inhibitor was approved by the FDA in November 2023 for treating HR-positive, HER2-negative metastatic breast cancer with at least one alteration in PIK3/CA/AKT1/PTEN genes, in combination with fulvestrant [62,63].

In general, PI3K-AKT inhibitors are prone to side effects, especially hyperglycemia (high blood sugar), which occurs in up to 80% of patients in clinical trials [64]. Other side effects of PI3K-AKT inhibitors include diarrhea, colitis, nausea, and rashes [64,65,66]. Thus, further developments in the field of PI3K-AKT inhibitors are needed to mitigate these debilitating side effects.

Similar to the PI3K/AKT pathway, many inhibitors have been developed to target the RAS/RAF/MEK pathway. Two examples are Sorafenib, which mainly inhibits RAF but has since been shown to inhibit other targets as well, and Trametinib, an MEK inhibitor [67]. One of the challenges of targeting this pathway is the prevalence of mutations; however, a large breakthrough in this area occurred in 2022 with the development of a covalent inhibitor of KRAS(G12C) [68]. It has been found that RAF inhibitors combined with MEK inhibitors give the most promising clinical results, but drug resistance remains a large problem in this field of inhibitor development [69].

Regarding the JAK/STAT signaling pathway, STAT3 stands out as a therapeutic target since it is downstream of the JAK proteins, which means targeting STAT instead of JAK should cause less off-target side effects. Additionally, STAT3’s involvement in promoting tumorigenesis and resistance to therapy also supports the hypothesis that targeting STAT3 is a promising therapeutic approach for cancer therapy [1,20]. STAT3 modulates numerous pathways critical for tumor survival, including apoptosis inhibition, immune evasion, angiogenesis, and EMT [21,29]. Therefore, inhibiting STAT3 could provide multifaceted benefits by disrupting these cancer-supportive mechanisms simultaneously. First of all, due to its role as a transcription factor, targeting STAT3 also inhibits downstream oncogenic gene expression including targets such as c-Myc, cyclin D1, and survivin. By targeting STAT3, these downstream oncogenic pathways can be suppressed, potentially reducing cell proliferation and inducing apoptosis across various cancer types.

STAT3 not only drives oncogenic signaling but also contributes to chemoresistance, a major obstacle in cancer therapy. In TNBC and esophageal squamous cell carcinoma, for instance, STAT3 activation has been correlated with resistance to chemotherapy agents mediated by STAT3’s regulation of anti-apoptotic proteins such as Bcl-2 and Bcl-xL [70]. Similarly, in head and neck cancers, STAT3 signaling confers resistance to platinum-based therapies, diminishing the efficacy of standard treatments [71]. The association between STAT3 activation and chemoresistance highlights its importance as a therapeutic target, as inhibiting STAT3 could enhance cancer cell sensitivity to existing treatments. These findings indicate that STAT3’s oncogenic activities play a significant role in cancer progression, invasion, and metastatic potential, supporting its status as a prognostic marker and a therapeutic target.

STAT3 plays a significant role in shaping the immunosuppressive tumor microenvironment (TME), contributing to immune evasion by upregulating immune-suppressive cytokines (e.g., IL-10, TGF-β) and downregulating pro-inflammatory cytokines [72]. Inhibiting STAT3 could restore immune function within the TME, making it more susceptible to immune-mediated cell death [73].

Additionally, STAT3 is critical for the maintenance of cancer stem cells (CSCs), which are implicated in cancer recurrence and metastasis. In cancers such as glioblastoma and pancreatic cancer, STAT3 activation supports CSC properties, including self-renewal and resistance to conventional therapies [10,74,75,76]. Targeting STAT3 in these cells could reduce tumor recurrence and improve patient outcomes. For example, the small-molecule STAT3 inhibitor napabucasin has been shown to attenuate the tamoxifen resistance of breast cancer cells by reducing the CSC properties [77].

Finally, STAT3 inhibitors are attractive therapeutics because STAT3 inhibition have been shown to synergize with other therapeutic modalities, such as immunotherapy, chemotherapy, and target therapies, to enhance their effectiveness [58,78,79]. Combining STAT3 inhibitors with immune checkpoint inhibitors, for example, has shown promise in preclinical studies, where STAT3 inhibition sensitizes tumors to immune cell infiltration and attacks [80].

## 4. Evaluation of STAT3 Inhibitors of Y705 and/or S727 in Clinical Development

**Figure 2:**
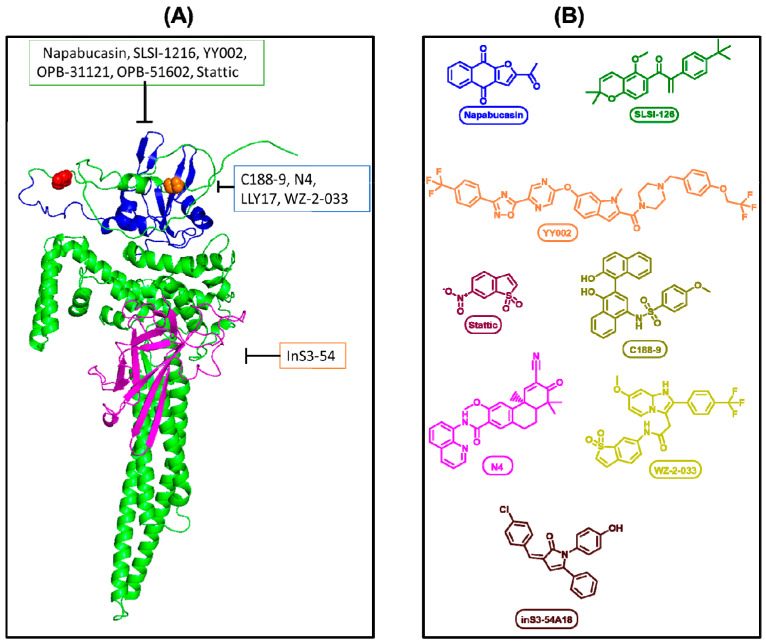
(**A**) Representation of drug target sites. Single STAT3 protein, folding performed by AlphaFold (AF-P40763-F1-v4). Blue region indicates Src Homology 2 domain (SH2). Magenta indicates DNA binding domain. Red spheres indicate Ser727 residue. Orange spheres indicate Tyr705. (**B**) Chemical structures of the representative inhibitors.

Stattic was the first developed STAT3 inhibitor and prevents STAT–STAT dimerization; however, its high toxicity prevented it from progressing to clinical trials of any kind [81]. Despite the diversity of STAT3 inhibitors developed to date, many candidates have shown poor bioavailability, structural instability, and off-target toxicity upon reaching clinical trials (Figure 2) [9,82,83,84,85].

Napabucasin (BBI608) is a small-molecule STAT3 inhibitor that has been shown to reduce breast cancer [86,87] and glioblastoma [75] progression by mediating STAT3 signaling. Napabucasin has been said to bind to the STAT3 SH2 domain, but there is currently a lack of definitive crystallographic evidence [88]. However, napabucasin has been shown to block both pY705 and S727 [77]. Napabucasin was granted orphan drug status in 2016 by the FDA for gastric cancer while also being explored for several other cancers [89]. A Phase Ib/II study in TNBC showed that napabucasin combined with paclitaxel was well tolerated and showed promising results which indicated anticancer activity [87]. Conversely, napabucasin was discontinued in Phase III clinical trials for pancreatic cancer due to futility with no additional safety concerns listed [90].

SLSI-1216 was developed to target STAT3 in TNBC and has been shown to affect the SH2 domain, specifically inhibiting EMT and reducing both pY705 and pS727 [30]. However, SLSI-1216 has not yet progressed to clinical trials.

C188-9 (TTI-101) has been studied as a STAT3 inhibitor in head and neck squamous cell carcinoma and specifically inhibits pY705 to reduce migration and invasion, as well as increase chemosensitivity in vitro [82]. C188-9 targets the SH2 domain and has been shown to sensitize pancreatic cells to 5-Aza-2′-deoxycytidine (DAC) treatment in mice [91]. Activity against pY705 has been measured in acute myeloid leukemia, and there is little evidence for the effect on pS727 [92]. C188-9 has been studied in a Phase 1b/2 clinical trial in advanced hepatocellular carcinoma in which it was well tolerated and displayed clinical benefits in about 60% of patients as a monotherapy [93].

InS3-54 is a small-molecule STAT3 inhibitor that targets the DNA-binding domain; as such, it does not inhibit pY705 activation or total STAT3 expression but reduces STAT3 activity by disrupting DNA binding, leading to the decreased expression of downstream genes [94]. N4 targets the STAT3 SH2 domain in pancreatic cancer and selectively blocks pY705 over pS727 [95]. N4 effectively inhibits STAT3 dimerization, as well as crosstalk with EGFR and NF-κB, and animal models have shown that N4 was well tolerated and effectively suppressed tumor growth and metastasis. There are currently no clinical trial data available for N4.

YY002 is another small-molecule therapeutic for pancreatic cancer that targets the STAT3 SH2 domain but, unlike N4, it blocks both pY705 and pS727 [96]. This dual blockage disrupts both the nuclear and mitochondrial functions of STAT3. YY002 displays good pharmacokinetics and an acceptable safety profile, as well as better antitumor efficacy compared to napabucasin [96]. Specifically, YY002 has been found to reduce TNBC and pancreatic tumor volumes at a lower dosage compared to napabucasin.

LLY17 is a small-molecule inhibitor developed for TNBC, targeting the STAT3 SH2 domain and decreasing pY705; however, pS727 was not examined in the initial study on this inhibitor [97]. The small-molecule inhibitor WZ-2-033 has been studied in TNBC and gastric cancer; it targets the STAT3 SH2 domain and specifically affects pY705 but not pS727 [18]. Currently, WZ-2-033 is still in the preclinical phase. Another small molecule inhibitor, OPB-31121, interacts with the STAT3 SH2 domain and decreases both pY705 and pS727 [98]. A Phase 1 clinical trial has been completed, but the inhibitor was considered to have failed in subjects with advanced solid tumors due to adverse effects and therapeutic resistance [99].

OPB-51602 is a small-molecule inhibitor that binds to the SH2 domain and disrupts mitochondrial STAT3 [100]. Both pY705 and pS727 were found to be inhibited at nanomolar concentrations [22]. OPB-51602 was tested in a Phase I clinical study of non-small-cell lung cancer, and the results showed promising antitumor activity coupled with a poor tolerability of continuous dosing [19]. A separate Phase I study in hematological malignancies displayed no clear therapeutic response, and while it was well tolerated at low dosages, long-term administration at higher dosages produced adverse effects [101].

Bayer’s orally available STAT3 inhibitor (VVD-130850) is currently being tested in Phase I clinical trials in conjunction with or without a checkpoint inhibitor, Pembrolizumab, in hematologic tumors [102]. VVD-130850 binds to an allosteric pocket of STAT3, which prevents STAT3 binding to the target genes.

A unique type of STAT3 inhibitor is Novo Nordisk’s small interfering RNA inhibitor, DCR-STAT3 [103]. Since this inhibitor ubiquitously knocks down STAT3, it affects both pY705 and pS727. DCR-STA3T is currently being evaluated in a first-in-human study to complete a safety and tolerability assessment in adults with refractory solid tumors. A benefit of this type of inhibitor is that it also disrupts unphosphorylated STAT3 (U-STAT3), which has also been shown to play an oncogenic role in STAT3 signaling [104,105,106]. U-STAT3 is capable of binding to DNA and thus regulating transcription, similar to phosphorylated STAT3 [105]. Additionally, U-STAT3 has also been shown to indirectly affect NF-kB-regulated gene expression by binding to NF-kb and regulating its nuclear transport, aiding in the constitutive activation of NF-kB and promoting oncogenic progression [107]. Thus, especially in cancer types with hyperactive NF-kB, it may be necessary to fully remove STAT3 in order to combat the U-STAT3 oncogenic pathways.

In addition to small interfering RNA inhibitors like DCR-STAT3, another novel approach to STAT3 inhibitor design that mitigates U-STAT3 signaling effects is proteolysis-targeting chimeras (PROTACs). PROTACs offer unique advantages over the traditional small-molecule inhibitors with respect to difficult drug targets because they can target any part of the protein for degradation rather than a specific binding pocket [108]. Utilizing PROTACs may improve STAT3 inhibitor efficacy due to the mechanism of action which allows for PROTAC molecules to be reused within the cell following protein degradation by the cell’s own proteasomal system [109].

Overall, STAT3 inhibitor development has encountered many challenges, especially with respect to clinical translation. Despite the diversity of STAT3 inhibitors developed to date, many candidates have shown poor bioavailability, structural instability, and off-target effects upon reaching clinical trials, in addition to therapeutic resistance and ineffectiveness [9,82,83,84,85].

Based on the inhibitors discussed above, toxicity is one of the greatest challenges faced in STAT3 inhibitor development. This observation is not unsurprising given that it has been shown that STAT3 is a necessary gene for embryonic development in mice, indicating that STAT3 plays crucial regulatory roles in regular cell growth and homeostasis [7]. Thus, any inhibitor of nuclear STAT3 risks targeting healthy cells in addition to cancer cells and inhibiting cell growth and proliferation. The global inhibition of STAT3 negatively affects mitochondrial and transcriptional functions across cell types, causing severe side effects. Genini et al. showed that OPB-51602 caused mitochondrial dysfunction via accumulated STAT3 aggregates thus leading to cell death, and that this effect was increased under glucose starvation conditions [22]. However, this demonstrated toxicity via mitochondrial STAT3 could occur in healthy cells as well. One consideration for mitigating these toxicities is to lower the effective concentration needed to reduce the effect on healthy cells. However, this approach must not sacrifice overall drug efficacy. Another possible way of addressing these toxicities is to improve the cancer cell-specific targeting of STAT3 inhibitors, such as through the use of antibody–drug conjugates. Antibody–drug conjugates allow for more the specific targeting of cancer cells through an antibody targeting a cancer cell-specific antigen in order to deliver the drug needed to kill the cancer cells, such as a STAT3 inhibitor [110].

While significant progress has been made with respect to STAT3 inhibitors for cancer applications, more mechanistic data are needed on the role of STAT3 in cancer in order to improve the translation of pre-clinical STAT3 inhibitors to clinically meaningful therapeutics.

## 5. Comparative Analysis: Targeting Y705 vs. S727 in STAT3 Inhibitor Design

As discussed in Section 2, downstream STAT3 signaling is influenced by both pY705 and pS727. In general, the inhibition of pY705 leads to decreased STAT3 transcription in the nucleus, while pS727 inhibition reduces mitochondrial STAT3 activity [9,57]. Additionally, there have been mixed results as to whether these pathways occur independently or concurrently with one another [15,16,17]. Thus, further research is needed to understand the mechanistic outcomes of pY705 and pS727 independently and concurrently in order to better understand STAT3 inhibitor design with respect to these two residue targets.

Based on the results detailed in Section 4, compounds that specifically target pY705 such as N4 and WZ-2-033 have been well tolerated in preclinical studies and have also shown good efficacy in preclinical models, but there is a lack of evidence suggesting effective clinical applications (Table 2). It is unclear whether some small molecules, such as C188-9 and LLy17, depend on inhibiting both the phosphorylation sites or only the tyrosine residue due to lack of experimental investigation of pS727. If C188-9 is pY705 specific, then there is evidence of clinical benefits in about 60% of patients with advanced hepatocellular carcinoma.

In general, compounds targeting pY705 have shown the most clinical success as far as efficacy is concerned, potentially due to the fact that compounds targeting pS727 tend to show increased toxicity. For instance, napabucasin, which is known to inhibit both phospho-sites, and C188-9, which may or may not inhibit pS727 in addition to pY705, have been well tolerated in clinical trials. Contrastingly, OPB-31121, which is known to inhibit both phospho-sites, was discontinued due to adverse effects. Each of these compounds have displayed some promising antitumor activity, with C188-9 being the only one to show high clinical efficacy.

## 6. Conclusions

Our analysis highlights the critical need for preclinical studies to investigate the effects of potential therapeutics on both pY705 and pS727. Without such data, it remains challenging to compare inhibitors across the various stages of development and fully evaluate the advantages and disadvantages of targeting each residue. These foundational mechanistic insights would be instrumental in predicting both toxicities and therapeutic effectiveness as the repertoire of STAT3 inhibitors expands.

Current evidence suggests that while pS727 plays a pivotal role in mitochondrial STAT3 translocation and cancer progression, the excessive inhibition of pS727 could lead to undesirable toxic effects in clinical trials. Based on these findings, we propose that future STAT3 inhibitor development should prioritize the small molecules targeting pY705 to minimize off-target toxicity. However, the most effective inhibitors might combine selective pY705 inhibition with moderate pS727 inhibition (at a higher IC50) to harness the therapeutic benefits of suppressing mitochondrial STAT3 activity while maintaining a favorable safety profile.

In addition, future therapeutic strategies should consider the role of unphosphorylated STAT3, particularly in cases where blocking pY705 and/or pS727 proves insufficient for achieving meaningful clinical outcomes. Exploring alternative approaches, such as small interfering RNA (siRNA), could be advantageous if these technologies can be made cancer specific to minimize the effects on healthy cells.

While STAT3 is well established as a promising therapeutic target across multiple cancer types, significant gaps in our understanding remain. These include differences in cancer-specific STAT3 signaling and post-translational modifications. Addressing these gaps is essential for designing safe and effective STAT3 inhibitors that can achieve clinical success.

## Figures and Tables

**Table 1:** List of tumor types with activated STAT3.

Tumor Type	Reference(s)
Breast cancer	[3,4,11,44,45]
Triple-negative breast cancer	[30,43]
Gastric cancer	[18]
Lung cancer	[46,47]
Glioblastoma	[48,49,50]
Hepatocellular carcinoma	[51,52]
Pancreatic cancer	[53,54]
Prostate cancer	[55]
Colorectal cancer	[56]
Esophageal cancer	[57,58]

**Table 2:** List of STAT3 inhibitors including the target domain, affected phospho-residues, clinical efficacy, toxicity, and current status of clinical phase trials. PY705 targets are associated with nuclear transcriptional function while pS727 targets are associated with mitochondrial function. With the exception of DCR-STAT3, which interacts with STAT3 RNA, and InS3-54 which blocks DNA binding in the nucleus, these inhibitors interact with STAT3 in the cytoplasm to prevent nuclear and/or mitochondrial translocation.

Compound	Target Domain	pY705	pS727	Clinical Efficacy	Toxicity	Current Status
Stattic	SH2	Yes	Yes	N/A	High	
Napabucasin (BBI608)	SH2	Yes	Yes	Low	Low	Phase I/II
SLSI-1216	SH2	Yes	Yes	N/A	N/A	
C188-9 (TTI-101)	SH2	Yes	N/A	High	Low	Phase 1/II
InS3-54	DNA- binding	No	No	N/A	N/A	
N4	SH2	Yes	No	N/A	Low	
YY002	SH2	Yes	Yes	N/A	Low	
LLY17	SH2	Yes	N/A	N/A	N/A	
WZ-2-033	SH2	Yes	No	N/A	Low	
OPB-31121	SH2	Yes	Yes	Low	High	Phase I
OPB-51602	SH2	Yes	Yes	High	High	Phase I
VVD-130850	Allosteric	N/A	N/A	N/A	N/A	
DCR-STAT3	RNA	Yes	Yes	N/A	N/A	

## Data Availability

No new data were created or analyzed in this study. Data sharing is not applicable to this article.

## Abbreviations

The following abbreviations are used in this manuscript:

CSC	Cancer stem cell
EGF	Epidermal growth factor
EMT	Epithelieal-to-mesenchymal transition
IL-10	Interleukin-10
IL-6	Interleukin-6
JAK	Janus kinase
MAPK	Mitogen-activated protein kinase
MitoSTAT3	Mitochondrial STAT3
PROTAC	Proteolysis-targeting chimeras
ROS	Reactive oxygen species
S727	Serine-727
Src	Proto-oncogene tyrosine-protein kinase Src
STAT3	Signal transducer and activator of transcription 3
U-STAT3	Unphosphorylated STAT3
VEGF	Vascular endothelial growth factor
Y705	Tyrosine-705
